# Discrepancies between farmers' perceptions and actual animal welfare conditions on commercial pig farms

**DOI:** 10.3389/fvets.2022.1010791

**Published:** 2022-09-29

**Authors:** Eva Nadlučnik, Irena Golinar Oven, Iztok Tomažič, Jan Plut, Alenka Dovč, Marina Štukelj

**Affiliations:** ^1^Veterinary Faculty, University of Ljubljana, Ljubljana, Slovenia; ^2^Clinic for Ruminants and Pigs, Clinic for Reproduction and Large Animals, Veterinary Faculty, University of Ljubljana, Ljubljana, Slovenia; ^3^Biotechnical Faculty, University of Ljubljana, Ljubljana, Slovenia; ^4^Clinic for Birds, Small Mammals and Reptiles, Veterinary Faculty, University of Ljubljana, Ljubljana, Slovenia

**Keywords:** animal welfare, commercial pig farms, farmers' perceptions, human-animal relationship, education

## Abstract

Animal welfare is a multiparameteral concept that encompasses the physical and mental health of animals and includes various aspects such as physical wellbeing, absence of hunger and thirst, and ability to express motivated behavior, to which farmers usually attach different importance. The objectives of this study were to evaluate animal welfare on Slovenian commercial pig farms, to determine whether farmers' perceived importance of animal welfare differ from actual animal welfare on farms and to determine, if farmer's age, gender, their level of education and participation in vocational training have an influence. For that purpose, we created an Animal Welfare Protocol/Questionnaire for Pig Farms (AWQ/P-P) that assessed several parameters of animal welfare: (1) general status, (2) animal behavior, (3) health status, (4) living conditions, and (5) environmental conditions. Each parameter included at least five observation points and was scored on a 5-point scale. The same observation points were used to measure farmers' perceived importance of animal welfare and for observational assessment. Consequently, we were able to compare both statistically. Farmers from 14 (*N* = 14) large Slovenian pig farms participated in the study. Results show that farmers rate all parameters of animal welfare very highly. For them, animal health status is the most important, and environmental conditions are the least important factors for animal welfare. Observational inspections yielded significantly lower scores for animal welfare conditions than those obtained from farmer ratings. The highest correlations between farmers' perceptions and observational inspections were found for the parameters of animal behavior and environmental conditions. The results of this study also suggest that vocational training is a significant variable in increasing levels of pig welfare. Age, gender, and education level are not significant variables, however, farms led by older male farmers with lower level of education but involved in vocational training from different sources had slightly better welfare on the farm. This should be further investigated before making conclusions, due to our small sample size. The significance of the study is to identify deficiencies in pig welfare as perceived by farmers and consequently improve pig welfare.

## Introduction

Slovenian pig farms are small and fragmented, agricultural land is limited, and natural conditions are not favorable for a larger scale of pig breeding (1). Pig farming makes up a small part of Slovenian agriculture, as the self-sufficiency rate for pork is only 20–25%. There are a total of 253,770 pigs in Slovenia, 22,262 of which are breeding sows. Pigs are bred on 12,843 farms, classified as commercial, non-commercial, and outdoor pig production. Only 22 of the farms are considered large with more than 1,000 pigs, the rest of the farms are small. Eleven thousand six hundred and thirty-one farms have 20 or fewer pigs (2).

Animal welfare is a broad term and can be defined in several ways, many of which are covered by the well-known five freedoms based on Brambell Commission's report to enquire into the welfare of animals kept under intensive livestock husbandry systems and created by the Farm Animal Welfare Council in 1979 (3, 4). World Organization of Animal Health declared in its Introduction to the recommendations for animal welfare, that “animals experience good welfare if they are healthy, comfortable, well-nourished, safe, are not suffering from unpleasant states such as pain, fear and distress, and can express behaviors that are important for their physical and mental state” (5).

Recently, three fundamental scientific concepts and approaches to the study of welfare have been developed worldwide. The first concept connects animal welfare to their natural environment (4, 6–8). Animals should live in an environment that allows them to behave in a natural way (9). Pigs are strongly motivated to express natural behaviors such as rooting, nesting and exploring, and in the impoverished environment they generally encounter in intensive breeding systems, they direct this natural need toward what is available to them—the equipment of the pen and other pigs (10, 11). The resulting behaviors, performed without an apparent function, are referred to as stereotypies and manifest as bar biting, sham chewing, and tongue rolling (4, 12). Stereotypies are therefore a clear indicator of impaired welfare (4, 13). Pigs are social animals that establish hierarchy in groups through aggression. However, aggression is absent in stable groups but does occur when pigs mix, encounter unfamiliar animals, or when resources are limited (1, 11).

The second concept of wellbeing links wellbeing to the biological functioning of animals. A central question in this concept is how an animal adapts to different environment (4, 8). Indicators of wellbeing regarding the environment primarily include an assessment of the animal health status, injuries, behavioral measurements, and quantitative measurements of physiological values such as cortisol levels (14). Production parameters have been considered an appropriate measure of welfare and low productivity an indicator of a lower welfare standard by scientists and farmers (15, 16). However, highly productive pigs can be mentally compromised (4), because they are often subjected to stress, which is a result of the desire for the greatest possible economic return (9), even though they may successfully adapt to such environment (14). From the pig's perspective, the environment in which it lives includes temperature, humidity, access to feed and water, and air quality (4). Among the environmental conditions, the temperature is the most important for the welfare of pigs, as they are highly susceptible to heat stress (1).

The third concept refers to the subjective feelings or affective states (4, 6–8). The feelings are negative with negative subjective states, such as hunger, thirst, pain, fear, and frustration, and positive with positive states, such as comfort and satisfaction with certain social interactions (4, 6). Reimert et al. conducted a study on pig behavior and cited tail wagging, play behavior and “play” bark vocalization as indicators of positive emotions in pigs (17). Many studies have demonstrated the importance of a positive human-animal relationship in reducing stress and enabling high productivity in farm animals (13, 18–22). Unpleasant handling, such as physical force, using electric shock and shouting negatively affects animals' health, productivity, behavior, welfare (4, 23, 24) and reduces meat quality (18). Zupan et al. examined the effects of early human handling on play and exploratory behavior in pigs and found that positive gestures prior to weaning, such as gentle petting on the back affected play behavior, object-oriented exploration, and the latency to approach a novel object or environment after weaning (22). Muns et al. discovered that positive human contact shortened the duration of piglet's escape behavior to tail docking, reduced the pigs' fear of humans and modified the behavioral responses to stressors (20).

The objectives of this study were (i) to evaluate animal welfare on commercial pig farms, (ii) to determine whether farmers' perceptions of animal welfare differ from actual animal welfare on farms, (iii) to determine, if farmer's age, gender, their level of education and whether they participate in vocational training (informal training, i.e., conferences, reading professional literature in their field, etc.) have an effect on welfare on the farm or on farmer's perception of animal welfare.

## Materials and methods

### Farms and farmers

Fourteen commercial pig farms and farmers participated in the study (Table 1). Half of the participants were female. Four were under 40 years of age. Nine of them had completed high school, the others had higher education (higher vocational school or university). All participants were taking part in vocational training from different sources: reading professional journals and books, attending congresses and lectures, collaborating with the experts and their own projects on the farm (projects that contribute to better welfare such as building bigger nursery pens, modernizing feeding technology with electronic sow feeders, etc.). Farmers differed from each other in terms of the number of sources from which they receive vocational training (Table 2).

**Table 1:** Characterization of the visited pig farms (*n* = 14).

**Farm characteristics**		**Number of farms**
Type of production	Farrow to finish	11
	Rearing weaners up to 30 kg	3
Housing system	Indoor	6
	Indoor with outdoor access	7
	Outdoor	1
Number of pigs	< 100	1
	101–500	7
	501–1,000	4
	>1,000	2
Breeding other farm animals	Pig farming only	7
	Poultry	5
	Wild ruminants	2

**Table 2:** Characterization of farmers' education and vocational training.

**Level of education**	**2 or less sources of vocational training**	**3 or more sources of vocational training**	**Total**
Completed only high school	4	5	9
Completed or enrolled in a higher vocational school or university	1	4	5
Total	5	9	14

### Protocol

For this study, an Animal Welfare Protocol/Questionnaire for Pig Farms (AWQ/P-P) that assessed several parameters of animal welfare was established: (1) general status—five parameters, (2) animal behavior—six parameters, (3) health status—eight parameters, (4) living conditions—five parameters, and (5) environmental conditions—six parameters. The animal welfare assessment protocol and the questionnaire of farmers' perceived importance of animal welfare on their farms (self-assessment) were identical in content and were used to compare farmers' perceived importance of animal welfare in pig farming with actual conditions on their farms. AWQ/P-P is included as Supplementary material S1. Observational assessment parameters were scored on a 5-point scale, while farmers' perceived importance was scored by 5-point Likert scale.

The welfare protocol was always assessed by two observing veterinarians. To minimize the differences between the two and to standardize the scores from the visits, observers received identical training prior to the assessment. The importance of the values in observational assessment is as follows: (1) major deficiencies (immediate action required), (2) deficiencies warranting a warning, (3) minor deficiencies (advice required), (4) no deficiencies (compliant with standards), and (5) no deficiencies (above-standard conditions). For each observation points, additional descriptions were provided (Supplementary material S1). The legal norm for setting up the points scale was “Rules on the protection of farm animals” from the Official Gazette of the Republic of Slovenia, Nos. 51/10 and 70/10 (25).

Questions in the questionnaire measuring farmers' perceived importance of animal welfare began with “In your opinion, how important is...?” (e.g., “How important do you think it is that lighting on the farm is not too strong or too weak, too short or too long?”). The scale represents the level of importance to farmers: (1) not important at all, (2) not important, (3) undecided, (4) it is important, (5) it is very important. In addition, several independent variables were included in the instrument: respondents' age and gender, their level of education, and whether they participate in vocational training.

First, the welfare of breeding sows, growers, and finishing pigs was assessed using the protocol, followed by an interview with the farm owner about his views on the welfare, using a questionnaire. Farms were visited during the period from July 9, 2021 to October 27, 2021.

### Statistical analyses

All raw data were first transferred to MS Excel and transformed for use in SPSS (ver. 26). Mean values were calculated for each parameter of the questionnaire (general status, animal behavior, health status, living conditions, and environmental conditions) and compared using the independent variables—age, gender, level of education, and vocational training (Mann-Whitney test). In addition, the Wilcoxon test was applied to compare the observational results with farmers' importance of welfare ratings for individual parameters from the protocol and questionnaire. For each parameter, Spearman's correlation coefficients between observational results and farmers' perceived importance of animal welfare were calculated. Due to the small sample size, effect sizes were calculated to determine the strength of the statistical differences using the formula $r=Z/\sqrt{N}$. Values < −0.2 or > 0.2 were treated as significant.

## Results

### Animal welfare on commercial farms

On observational assessment, the highest score was achieved for the parameter animal behavior and lowest for living conditions (Table 3). See Supplementary material S2 for full results with all the parameters and Wilcoxon test. For four out of thirty parameters from the observational assessment, the average score was below 3.5. On the other hand, there were almost no scores above the standards. Only for the parameters B—observing the animals and C—presence of umbilical or inguinal hernias did the average scores reach values above 4.0, indicating above standard conditions in some farms.

**Table 3:** Comparisons between observational assessment and self-assessed importance of animal welfare.

**Parameters**	**Observational assessment**			**Self-assessed importance of welfare**			**Wilcoxon**		**Effect size**
	***M***	***SE***	***SD***	***M***	***SE***	***SD***	***Z***	***p***	***r***
General status (A)	3.7	0.16	0.60	4.6	0.09	0.35	−3.084	0.002	−0.82
Animal behavior (B)	3.9	0.12	0.46	4.4	0.11	0.42	−2.947	0.003	−0.79
Health status (C)	3.8	0.08	0.30	4.8	0.07	0.25	−3.306	0.001	−0.88
Living conditions (D)	3.6	0.12	0.43	4.5	0.11	0.40	−3.188	0.001	−0.85
Environmental conditions (E)	3.7	0.09	0.32	4.3	0.13	0.50	−2.981	0.003	−0.80

O, observational assessment; S, self-assessed importance of animal welfare; M, Mean; SE, Standard error; SD, Standard deviation.

### Comparisons between observational assessment and self-assessed importance of animal welfare

For all but one item (pigs' fear of humans), the experts' observations resulted in lower average scores than the participants' self-assessed data The highest self-assessed score was achieved for the parameter health status and the lowest for environmental conditions (Table 3; Supplementary material S2).

Correlations between observational and self-assessed scores for individual parameter show that there were significant correlations for animal behavior parameter only (Table 4). Medium correlations were also found for environmental conditions parameter, closing statistical significance. For both parameters, the higher the observational scores, the higher are scores from self-assessed importance values.

**Table 4:** Spearman's correlation coefficients between observational and self-assessed importance scores for individual parameter.

**Parameter**	***r_*S*_***	***p***
General status	−0.015	0.480
Animal behavior	0.524	0.027
Health status	0.168	0.283
Living conditions	0.205	0.241
Environmental conditions	0.414	0.071

### Effects of independent variables on observational assessment and self-assessed importance of animal welfare

In Table 5, there are effect sizes for individual independent variable presented. For full results with all the parameters and Mann–Whitney test see Supplementary material S3.

**Table 5:** Observational assessment and self-assessed importance of animal welfare effect sizes for gender, age, education level, and sources of vocational training.

**Categories**	**Effect sizes**			
	**Gender**	**Age**	**Education status**	**Sources of vocational training**
O_general status	0.00	−0.25	−0.23	−0.48
O_animal behavior	0.43	−0.10	−0.14	−0.27
O_health status	0.40	−0.26	−0.27	−0.13
O_living conditions	−0.03	−0.19	−0.20	−0.59
O_environmental conditions	−0.21	−0.21	−0.27	−0.22
S_general status	0.39	−0.08	−0.02	−0.09
S_animal behavior	−0.19	−0.40	−0.32	−0.41
S_health status	−0.39	−0.02	−0.51	−0.28
S_living conditions	−0.05	−0.73	−0.14	−0.31
S_environmental conditions	−0.24	0.10	0.00	−0.70

The results from the observational assessment show that female farmers scored lower on animal behavior and health status parameter and higher on environmental conditions parameter compared to male participants. There were no evident differences between the genders on general status and living conditions parameters. On the self-assessed importance of animal welfare, female farmers scored lower on health status and environmental conditions parameter and higher on the general status parameter than male farmers. There were no evident differences between the genders on living conditions parameter.

On farms where interviewed participants were older than 40 years, the scores from the observational assessment were higher in the parameters of general status and environmental conditions, while they were lower in the parameter of health status than in farms where younger participants were interviewed. There were no evident differences in other parameters. On the self-assessed part, older participants scored higher in the parameters of animal behavior and living conditions than younger participants. In the latter parameter, a large difference was found between age groups. There were no evident differences in other parameters.

Depending on education level, differences were found in four out of five parameters of observational assessment. Participants with lower educational level scored higher on general status, living conditions and environmental conditions parameters. In contrast, higher education level participants scored higher on the health status parameter. On the self-assessed part, it is evident that participants with higher education level rate health status higher while they rate animal behavior lower than the participants with lower education level.

Participation in various sources of vocational training affected four out of five parameters of observational assessment. Namely, participants who train from more sources scored higher than participants who train in fewer sources of vocational education. The same was true for four out of five parameters of the assessed importance of animal welfare.

## Discussion with conclusions

Compared with preceding studies addressing farmers' perceptions of animal welfare this study also presents general information about commercial pig farms in Slovenia and the effect of different variables on animal welfare. The observers' evaluation showed that animal welfare in commercial pig farms in Slovenia can generally be scored as positive. As mentioned earlier, in only 4 out of 30 observation points, the average score was below 3.5, which means that advice should be given on these issues to improve animal welfare conditions. These observation points were: biosecurity on farms, lack of appropriate enrichment materials, no separation of pigs by different categories, and the lack of thermometers and hygrometers on farms. However, the farms reflected only compliance with the minimum requirements. Only in two observation points (pigs' fear of humans and the presence of hernias), farms reached above the average score. Farms had the highest welfare status regarding animal behavior (pigs not fearing humans, less aggression and fights among pigs, pigs showing curiosity, etc.) and lowest regarding living conditions of the pigs (stocking density, feeding space, enrichment material, etc.). Our results are similar to those from the study of Golinar Oven et al. on animal welfare in Slovenian conventional and alternative pig production systems using WQ^®^ protocol (12). The conclusion was that growers and fatteners in Slovenian conventional farms were rated as acceptable, but Slovenian alternative farms were rated as enhanced.

The study shows that there are discrepancies between actual animal welfare on selected farms and farmers' self-assessed importance of animal welfare. With one exception (pigs' fear of humans and its importance), the experts' observations resulted in lower average scores than the participants' self-assessment. Slovenian farmers rate all parameters of animal welfare very highly. Many studies which include participants from different countries of the world reported similar results—people generally find animal welfare and the laws that protect animals important (26), most people want better welfare for animals (27), and find animal protection an important social issue (28). Animal health status is the most important, and environmental conditions are the least important factors for animal welfare, according to farmers in our study. Similar to our results, participating farmers in a study from Vigors et al. selected minimizing health issues as the most important factor for animal wellbeing (29). We also discovered that the farmers who rate animal behavior as the most important also have better actual welfare on the farms, regarding the parameter. Kiliç and Bozkurt conducted a similar study on the relationship between farmers' perceptions and animal welfare standards on sheep farms and found that farmers who rated the importance of welfare higher, had better actual welfare on their farms (30), similar results were reported by Munoz et al. who studied the relationship between farmer attitudes, management behavior and sheep welfare (31). Albernaz-Gonçalves et al. identified numerous management and animal indicators of poor welfare on the farms, included in their study. However, most farmers surveyed were satisfied with animal welfare standards at their farms and were not willing to improve the status (15). Kauppinen et al. reported that farmers included in their study who considered improving animal welfare more important had higher productivity on their farms (19).

There are numerous studies examining farmers' motives and willingness to improve animal welfare (15, 19, 32–37). For many farmers worldwide, cost and investment are important motivators (15, 33, 35, 37–39). Additional welfare improvements on the farms in our study would mean greater expenditures that are not covered or subsidized by the government, so any additional costs fall on the shoulders of farmers. For instance, in the year 2021, the Decree on the animal welfare measure from the Rural Development Program of the Republic of Slovenia for the period 2014–2020 supported farms that met animal welfare requirements that went beyond minimum conditions and normal husbandry practices. Farms that had 10% more unobstructed floor space per animal in group pens according to minimum standards were supported by funding (40). This implies significant investments, especially if major infrastructure changes are required. Costs could be the reason farmers identify health status as the most important parameter of animal welfare, as health problems produce great expenses (41). Another farmers' important motivator for improving animal welfare is increasing productivity of the pigs, which is again related to higher income (4, 15, 33, 41).

The results of our study varied according to the independent variables. The results were clearest for the vocational training variable, where farmers who continue their education from multiple sources score higher on both actual welfare and farmers' perceived importance of welfare, on 4 of 5 parameters (general status, living conditions, and environmental conditions). This implies that vocational training contributes to better actual and self-assessed animal welfare. Jo et al. conducted a study on broiler farmers' perceptions of animal welfare and concluded that as education levels increase, farm productivity and efficiency also increase (42). Improved education leads to higher job satisfaction among farmers and positively affects their perception of animal welfare (32). Coleman et al. trained farm workers to test whether behavior and attitude toward pigs on a commercial farm can be altered. Not only was there a decrease in negative behaviors toward pigs, but the change in attitude also had a positive effect on pig behavior (34).

Interestingly, we discovered that farmers with lower education level had better welfare at their farms compared to farmers with higher education level, on 3 out of 5 parameters (general status, living conditions and environmental conditions). That indicates that the level of education is not as important as vocational training, especially engaging in different types of training. This contrasts with other researchers' studies, which have found a significant influence of farmers' higher education level on improving animal welfare (30, 31, 42). No significant relationship was found between educational level and self-assessed importance of welfare, as all the participants rated welfare highly. Participants with higher education levels found health status more important and animal behavior less important than the participants with lower education levels. As our study sample is small, we believe further investigation is necessary to determine the effect of education on pigs' welfare before making any conclusions.

The results suggest that age of the farmer has a slight impact on animal welfare. Older farmers' farms had pigs with better general status and the environmental conditions were better taken care of (dust, humidity, odors, ventilation, and heating). Younger farmers had better general health status of pigs (less problems with trotters, diarrhea, hernias, conjunctivitis etc.). Older farmers also find animal behavior and living conditions more important than younger participants which is interesting, as the actual welfare regarding those parameters did not differ from younger farmers. Studies that consider age as a variable for attitudes toward animal welfare are inconsistent. Some studies report, that older farmers had higher empathy scores and were more likely to intervene in pig fights than young farmers (32). Others did not find significant relationship between age and welfare (21, 30). Some studies concluded that younger farmers have better welfare status on their farms (36, 43). Jo et al. found that an increase in farmers' age decreases farming efficiency and production level by up to 0.16% (42).

Males achieved slightly better results than females in our study. If the farmer was male, pigs had better health status and pigs' behavior was better compared to female farmers. Females had better environmental conditions. Male farmers also find animal behavior, health status and environmental conditions more important than females. Females think general status is more important than males. This contrasts with previous studies which prove that female farmers, veterinarians, and veterinary students, on average, show higher levels of positive behavior and empathy toward animals (29, 32, 36, 44–49). However, the study by Kauppinen et al. did not find strong correlations between gender and welfare (19).

The small sample size is a major limitation of this study and presumably the reason why we found only one significant association. To make more relevant conclusions, we intend to broaden the sample through our project. On the other hand, we visited the majority of larger Slovenian farms considering that most of the Slovenian farms are small. We also intend to perform the same test on other farm animals (horses, poultry, and cattle) and compare the results to this study.

In conclusion, the pig farmers in Slovenia consider animal welfare very important, but their farms follow only minimal statutory requirements. The welfare on Slovenian farms is adequate, but there is room for improvement, especially regarding biosecurity on farms, lack of appropriate enrichment materials, no separation of pigs by different categories, and the lack of thermometers and hygrometers on farms. The results of this study also suggest that vocational training is a significant variable in increasing levels of pig welfare. Age, gender, and education level are not significant variables, however, we found slightly better welfare on farms led by older male farmers with a lower level of education, who enroll in vocational training from many sources. This should be further investigated before making conclusions, due to our small sample size.

To our knowledge, similar studies of discrepancies between farmers' perceptions and actual animal welfare conditions on any kind of pig farms have not yet been conducted. We believe that with this research we have opened a discussion in an important field that should be investigated further. This study was carried out within the framework of the Slovenian Target Research Program. The goal of the program is to adjust the welfare guidelines in Slovenia and to educate farmers on topics where we found irregularities on the farms and, as a result, to raise the level of welfare in Slovenian pig farms.

## Data availability statement

The raw data supporting the conclusions of this article will be made available by the authors, without undue reservation.

## Ethics statement

The evaluation of pig welfare in Slovenia was carried out within the framework of the Slovenian Target Research Program (Breeding of domestic animals by upgrading animal welfare in accordance with social requirements, No. V4-2024).

## Author contributions

MŠ, IT, and AD conceived and designed the study. EN, IGO, MŠ, and JP conducted the study. IT analyzed the data. EN wrote the manuscript. All authors reviewed and approved the final manuscript.

## Funding

The funding for this study was provided by Central Research Project (CRP V4-2024) and Research Core Funding (P4-0092) both financed by the Slovenian Research Agency (ARRS) and Ministry of Agriculture, Forestry and Food.

## Conflict of interest

The authors declare that the research was conducted in the absence of any commercial or financial relationships that could be construed as a potential conflict of interest.

## Publisher's note

All claims expressed in this article are solely those of the authors and do not necessarily represent those of their affiliated organizations, or those of the publisher, the editors and the reviewers. Any product that may be evaluated in this article, or claim that may be made by its manufacturer, is not guaranteed or endorsed by the publisher.

## References

[1] Kovač M Malovrh Š. Spremljanje proizvodnosti prašičev – IX. del. Domžale: Biotehniška fakulteta, oddelek za zootehniko, enota za prašičerejo. (2014). p. 87–105.

[2] Republic of Slovenia. Number of livestock. Ljubljana: Statistical Office of the Republic of Slovenia (2021).

[3] Brambell FWR Barbour DS Barnett MB Ewer TK Hobson A Pitchforth H. Report of the Technical Committee to Enquire Into the Welfare of Animals Kept Under Intensive Husbandry Systems. Her Majesty's Stationary Office: London (1965). p. 85.

[4] Johnson AK Colpoys JD Edwards-Callaway LN Calvo-Lorenzo M McGlone JJ Millman ST. Behavior and welfare. In:Zimmerman JJ Karrike LA Ramirez A Schwartz KJ Stevenson GW Zhang J, editors. Diseases of Swine. 11th ed. Hoboken, NJ: Wiley-Blackwell (2019). p. 17–41.

[5] World Organization of Animal Health. Introduction to the recommendations for animal welfare. In:World Organization for Animal Health (OIE), editors. Terrestrial Animal Health Code. Paris: World Organization for Animal Health (2008). p. 235–36.

[6] Carenzi C Verga M. Animal welfare: review of the scientific concept and definition. Ital J Anim Sci. (2009) 8(Suppl. 1):21–30. 10.4081/ijas.2009.s1.21

[7] Czychol I Büttner K Beilage E Krieter J. Review of the assessment of animal welfare with special emphasis on the “Welfare Quality^®^ animal welfare assessment protocol for growing pigs”. Arch Anim Breed. (2015) 58:237–49. 10.5194/aab-58-237-2015

[8] Fraser D. Animal welfare and the intensification of animal production. In:Thompson PB, editor. The Ethics of Intensification, Vol 16. Dordrecht, NL: Springer (2008). p. 167–89.

[9] Prunier A Heinonen M Quesnel H. High physiological demands in intensively raised pigs: impact on health and welfare. Animal. (2010) 4:886–98. 10.1017/S175173111000008X 22444261

[10] Machado SP Caldara FR Foppa L de Moura R Gonçalves LM Garcia RG. The behavior of pigs reared in enriched environment: alternatives to extend pigs attention. PLoS ONE. (2017) 12:e0168427. 10.1371/journal.pone.0168427 28060849 PMC5218552

[11] D'Eath RB Turner SP Marchant-Forde J. The natural behaviour of the pig. In:Marchant-Forde J, editor. The Welfare of Pigs. Dordrecht, NL: Springer (2009). p. 13–45.

[12] Golinar Oven I Štukelj M Plut J. Animal welfare assessment in Slovenian conventional and alternative pig production systems. Vet glasnik. (2021) 75:162–74. 10.2298/VETGL210930016G

[13] Hemsworth PH Barnett JL. The effects of aversively handling pigs, either individually or in groups, on their behaviour, growth and corticosteroids. Appl Anim Behav Sci. (1991) 30:61–72. 10.1016/0168-1591(91)90085-C

[14] Godyń D Nowicki J Herbut P. Effects of environmental enrichment on pig welfare-a review. Animals. (2019) 9:383. 10.3390/ani9060383 31234475 PMC6616547

[15] Albernaz-Gonçalves R Olmos G Hötzel MJ. My pigs are ok, why change? – animal welfare accounts of pig farmers. Animal. (2021) 15:100154. 10.1016/j.animal.2020.100154 33573976

[16] Hewson CJ. What is animal welfare? Common definitions and their practical consequences. Can Vet J. (2003) 44:496–9. 12839246 PMC340178

[17] Reimert I Bolhuis JE Kemp B Rodenburg TB. Indicators of positive and negative emotions and emotional contagion in pigs. Physiol Behav. (2013) 109:42–50. 10.1016/j.physbeh.2012.11.002 23159725

[18] Cornish A Raubenheimer D McGreevy P. What we know about the public's level of concern for farm animal welfare in food production in developed countries. Animals. (2016) 6:74. 10.3390/ani6110074 27854336 PMC5126776

[19] Kauppinen T Vesala KM Valros A. Farmer attitude toward improvement of animal welfare is correlated with piglet production parameters. Livest Sci. (2012) 143:142–50. 10.1016/j.livsci.2011.09.011

[20] Muns R Rault JL Hemsworth P. Positive human contact on the first day of life alters the piglet's behavioural response to humans and husbandry practices. Physiol Behav. (2015) 151:162–7. 10.1016/j.physbeh.2015.06.030 26130444

[21] Pol F Kling-Eveillard F Champigneulle F Fresnay E Ducrocq M Courboulay V. Human–animal relationship influences husbandry practices, animal welfare and productivity in pig farming. Animal. (2021) 15:100103. 10.1016/j.animal.2020.100103 33573972

[22] Zupan M Rehn T de Oliveira D Keeling LJ. Promoting positive states: the effect of early human handling on play and exploratory behaviour in pigs. Animal. (2015) 10:135–41. 10.1017/S1751731115001743 26290191

[23] Fraser D Duncan IJH Edwards SA Grandin T Gregory NG Guyonnet V. General Principles for the welfare of animals in production systems: the underlying science and its application. Vet J. (2013) 198:19–27. 10.1016/j.tvjl.2013.06.028 23899406

[24] Hemsworth PH Coleman GJ. Human-animal interactions and animal productivity and welfare. In:Hemsworth PH Coleman GJ, editors. Human-Livestock Interactions: The Stockperson and the Productivity and Welfare of Intensively Farmed Animals. 2nd ed. Wallingford: CAB International (2011). p. 47–83.

[25] Republic of Slovenia. Rules on the protection of farm animals. In: Republic of Slovenia. Ljubljana: The Official Gazette of the Republic of Slovenia (2011). No. 51/10 and 70/10.

[26] Sinclair M Lee NYP Hötzel MJ de Luna MCT Sharma A Idris M. International perceptions of animals and the importance of their welfare. Front Anim Sci. (2022) 3:960379. 10.3389/fanim.2022.96037931195720

[27] Anderson J Tyler L Attitudes Toward Farmed Animals in the BRIC Countries (2018). Available online at: https://faunalytics.org/wp-content/uploads/2018/09/BRIC-Full-Report.pdf (accessed August 31, 2022).

[28] Sinclair M Philips CJC. The cross-cultural importance of animal protection and other world social issues. J Agric Environ Ethics. (2017) 30:439–55. 10.1007/s10806-017-9676-5

[29] Vigors B Ewing DA Lawrence AB. The importance of farm animal health and natural behaviors to livestock farmers: findings from a factorial survey using vignettes. Front Anim Sci. (2021) 2:638782. 10.3389/fanim.2021.638782

[30] Kiliç I Bozkurt Z. The relationship between farmers' perceptions and animal welfare standards in sheep farms. Asian-Australas J Anim Sci. (2013) 26:1329–38. 10.5713/ajas.2013.13124 25049916 PMC4093410

[31] Munoz CA Coleman GJ Hemsworth PH Campbell AJD Doyle RE. Positive attitudes, positive outcomes: the relationship between farmer attitudes, management behaviour and sheep welfare. PLoS ONE. (2019) 14:e0220455. 10.1371/journal.pone.0220455 31365546 PMC6668801

[32] Balzani A Hanlon A. Factors that influence farmers' views on farm animal welfare: a semi-systematic review and thematic analysis. Animals. (2020) 10:1524. 10.3390/ani10091524 32872206 PMC7552314

[33] Borges JAR Domingues CHF Caldara FR Rosa NPD Senger I Guidolin DGF. Identifying the factors impacting on farmers' intention to adopt animal friendly practices. Prev Vet Med. (2019) 170:104718. 10.1016/j.prevetmed.2019.104718 31421489

[34] Coleman GJ Hemsworth PH Hay M Cox M. Modifying stockperson attitudes and behaviour towards pigs at a large commercial farm. Appl Anim Behav Sci. (2000) 66:11–20. 10.1016/S0168-1591(99)00073-8

[35] Hubbard C Bourlakis M Garrod G. Pig in the middle: farmers and the delivery of farm animal welfare standards. Br Food J. (2007) 109:919–30. 10.1108/00070700710835723

[36] Peden R Akaichi F Camerlink I Boyle L Turner S. Factors influencing farmer willingness to reduce aggression between pigs. Animals. (2018) 9:6. 10.3390/ani9010006 30583499 PMC6357189

[37] Schukat S Kuhlmann A Heise H. Fattening pig farmers' intention to participate in animal welfare programs. Animals. (2019) 9:1042. 10.3390/ani9121042 31795251 PMC6941093

[38] Fernandes JN Hemsworth PH Coleman GJ Tilbrook AJ. Costs and benefits of improving farm animal welfare. Agriculture. (2021) 11:104. 10.3390/agriculture11020104

[39] Molnár M Fraser D. Protecting farm animal welfare during intensification: farmer perceptions of economic and regulatory pressures. Anim Welf. (2020) 29:133–41. 10.7120/09627286.29.2.133

[40] Republic of Slovenia. Decree about the animal welfare measure of Rural Development Programme of the Republic of Slovenia 2014-2020 in the year 2021. In:Republic of Slovenia, editor. Official Gazette of the Republic of Slovenia (Ljubljana). (2021) No.3/21 and 28/21.

[41] Dawkins MS. Animal welfare and efficient farming: is conflict inevitable? Anim Prod Sci. (2007) 57:201–8. 10.1071/AN15383

[42] Jo H Nasrullah M Jiang B Li X Bao J. A Survey of broiler farmers' perceptions of animal welfare and their technical efficiency: a case study in Northeast China. J Appl Anim Welf Sci. (2021) 25:275–86. 10.1080/10888705.2021.1912605 33843378

[43] Peden R Camerlink I Boyle LA Loughnan A Akaichi F Turner SP. Belief in pigs' capacity to suffer: an assessment of pig farmers, veterinarians, students, and citizens. Anthrozoös. (2020) 33:21–36. 10.1080/08927936.2020.1694304

[44] Colombo ES Crippa F Calderari T Prato-Previde E. Empathy towards animals and people: the role of gender and length of service in a sample of Italian vets. J Vet Behav Clin Appl Res. (2017) 17:32–7. 10.1016/j.jveb.2016.10.010

[45] Bernuz Beneitez MJ María GA. Public opinion about punishment for animal abuse in Spain: animal attributes as predictors of attitudes toward penalties. Anthrozoös. (2022) 35:559–76. 10.1080/08927936.2021.2012341

[46] Mazas B Fernández Manzanal MR Zarza FJ María GA. Development and validation of a scale to assess students' attitude towards animal welfare. Int J Sci Educ. (2013) 35:1775–99. 10.1080/09500693.2013.810354

[47] Randler C Adan A Antofie MM Arrona-Palacios A Candido M Boeve-de Pauw J. Animal welfare attitudes: effects of gender and diet in university samples from 22 countries. Animals. (2021) 11:1893. 10.3390/ani11071893 34202129 PMC8300362

[48] Herzog HA. Gender differences in human–animal interactions: a review. Anthrozoös. (2007) 20:7–21. 10.2752/089279307780216687

[49] Bir C Davis M Widmar N Zuelly S Erasmus M. Perceptions of animal welfare with a special focus on Turkeys. Front Vet Sci. (2019) 6:413. 10.3389/fvets.2019.00413 31824971 PMC6881301

