# Supplementary material for: Discrepancies between farmers' perceptions and actual animal welfare conditions on commercial pig farms
Source: Front Vet Sci. 2022 Sep 29;9:1010791. doi: 10.3389/fvets.2022.1010791 (PMC9558291; doi:10.3389/fvets.2022.1010791)
Supplement: Supplementary file 1 [file Data_Sheet_1.pdf]

	**GENERAL INFORMATION** (personal data will stay anonymous)			**DATA**	
1	Date of the farm visit and the time spent on the farm.				
2	Name and surname of the expert questioner (1) and assistant (2)			**1**	**2**
3	Name and surname of the respondent ***he is the owner of the farm / he is a stock person***				
4	Type of husbandry and breed (hybrid, prominence...) of pigs on the farm				
5	Number of animals (by the type – suckling pigs, weaners, finishing pigs, boars, swine)				
6	Size of the usable floor area of the barn (m2) and height (m)				
7	Other farmed animals				
8	Types and number of individual categories of farm animals that are present on the farm (suckling pigs, weaners, finishing pigs, boars, swine)				

	**GENERAL INFORMATION** (personal data will stay anonymous)		**DATA**	
1	Year of birth		Gender	Male	female
2	Highest level of education achieved?	•	Primary school
		•	High school
		•	Grammar school
		•	Diploma (professional or post-secondary programs, 1st level)
		•	Master's degree (2nd level)
		•	Unified master's program
		•	Scientific master's degree
		•	PhD
3	What is your current employment?	•	Farmer
		•	Lodger, fisherman, hunter
		•	Craftsman, production worker
		•	Salesmen, other service professions
		•	Entrepreneur
		•	Office worker, official
		•	Employees in education, health care, engineer
		•	Senior official, manager
		•	Unemployed
		•	Retired
	If other, please specify	•	
4	What is your field of education?	•	Agricultural
		•	veterinary
	If other, please specify	•	

PIGS – A SPECIAL PART related to the viewing of the farm (completed by an expert during the husbandry tour)

1 - major deficiencies (immediate action required), **2** - deficiencies warranting a warning, **3** - minor deficiencies (advice required), **4** - no deficiencies (compliant with standards), and **5** - no deficiencies (above-standard conditions).

27		POINTS				
	**GENERAL IMPRESSION**					
1	Upon entering the stable, the animal breeder announces his arrival/speaks kindly.<br>***Ask the breeder to enter the farm as usual.***<br><br>0 - Uses completely inappropriate communication with the animals, screams, the pigs are visibly scared 1 – Does not communicate with the pigs, they are visibly scared 2 – Does not communicate with the pigs 3 – He/she kindly greets the pigs, 4 - He/she kindly greets the pigs, they approach him/her	1	2	3	4	5
2	(1) Before entrance to the stable, he has the sign "Entry prohibited"<br>(2) He has a barrier, which he also uses.<br>(3) Upon entering the stable, the biosecurity protection procedure is correct.<br>(4) The visit is also recorded in the guest book.<br><br>0 - does not practice any of the answers, 1 - at least 1, 2 - at least 2, 3 - at least 3, 4 - practices al	1	2	3	4	5
3	The water-throughs are leaking, the entire floor of the pens is dirty, the bedding is missing/worn out.<br><br>***Underline the appropriate answer***<br><br>0 - All three listed 1 - Two irregularities 2 - one irregularity from the above 3 – according to standards 4 - More water-throughs than according to standards, clean floor, flooring is suitable	1	2	3	4	5
4	Contaminated/moldy feed in troughs, feed on the ground next to feeders, questionable water quality.<br>***Underline the appropriate answer***<br><br>0 - All three of the above 1 - Two deficiencies 2 - One deficiency from the above 3 - According to standards, 4 - Feed and water quality is adequate, not loose, feeders and feeders are clean	1	2	3	4	5
5	Finishing pigs/weaned pigs are dirty, there are visible marks of aggressive behavior (wounds, scratches), tails and ears are visibly bitten/damaged.<br>***Underline the appropriate answer***<br><br>0 - More than 30% of animals have visible abnormalities, 1 - 25% of animals have visible abnormalities 2 - 10% of animals have visible abnormalities 3 - individual animals have visible abnormalities 4 - animals have no visible changes	1	2	3	4	5
	**ANIMAL BEHAVIOR**					
1	The animals are curious, and show exploratory behavior, no aggression is observed between the animals.<br>***Underline the appropriate answer***<br><br>0 - More than 30% of animals have visible abnormalities (apathetic / show signs of aggression), 1 - 25% of animals have visible abnormalities 2 - 10% of animals have visible abnormalities 3 - individual animals have visible abnormalities 4 - all animals are curious, show exploratory behavior, no visible aggression	1	2	3	4	5
2	The animals are visibly scared of the breeder, the caretaker, and strangers.<br>***Underline the appropriate answer***<br><br>0 - all animals become very agitated in the presence of a human and remain agitated for a long time 1 - all animals become visibly agitated in the presence of a human and eventually calm down, 2 - in individual pens the pigs become visibly agitated and eventually calm down 3 - animals become mildly agitated in the presence of a human and then they calm down quickly 4 - the animals do not get agitated in the presence of a person/breeder	1	2	3	4	5
3	The animals are crowded in one part of the pen due to inadequate temperature and/or draft.<br>***Underline the appropriate answer***	1	2	3	4	5

	0 - More than 30% of pens have pigs that are crowded on one part of the pen, 1 - 25% of pens have pigs that are crowded on one part of the pen, 2 - 10% of pens have pigs that are crowded on one part of the pen 3 – only a few smaller groups of pigs are crowded on one part of the pen, 4 - The animals are distributed throughout the pen, curious, lively					
4	Individual animals are limping and walking with difficulty.<br><br>0 - More than 30% of pigs limp /have difficulty walking, 1 - 25% of pigs limp /have difficulty walking, 2 - 10% of pigs limp / have difficulty walking 3 - Individual animals limp / have difficulty walking 4 - No pig limps / has difficulty walking	1	2	3	4	5
5	Animal handling is inappropriate (rough, unprofessional).<br><br>0 - The breeder pushes the pig off, kicks, and treats the pigs roughly 1 - When the breeder enters the pen, the pigs avoid him 2 - When the breeder enters the pen, the pigs avoid him, later they calm down 3 - When the breeder enters the pen, they the pigs do not panic, the individual pigs approach 4 - When the breeder comes to the pen, the animals immediately approach him	1	2	3	4	5
6	Do you watch animals during the day?<br>Together or several times a day for 10 minutes, 20 minutes, 30 minutes, 60 minutes.<br><br>0 - Does not observe animals during the day, 1 - Occasionally looks at animals during other tasks, 2 - Observes animals once a day, 3 - Observes animals twice a day, 4 - Observes animals several times a day	1	2	3	4	5
	**HEALTH STATUS**					
1	The animals are not equal in size and/or pigs are cachectic.<br>***Underline the appropriate answer***.<br><br>0 - More than 30% of pens have unequal/cachectic pigs present 1 - 25% of pens have unequal/cachectic pigs 2 - 10% of pens have unequal/cachectic pigs 3 - Individual unequal/cachectic pigs the farm 4 - All animals in pen are equal in size, pigs are in good body condition	1	2	3	4	5
2	Inspection of trotters and standing.<br><br>0 - More than 30% of animals have problems with trotters 1 - 25% of animals have problems with trotters 2 - 10% of animals have problems with trotters 3 - Individual animals have problems with trotters, 4 - No pig has problems with trotters	1	2	3	4	5
3	Coughing and/or sneezing is present<br>***Underline the appropriate answer***<br><br>0 - More than 30% of half pigs cough/sneeze 1 - 25% of pigs cough/sneeze 2 - 10% of pigs cough/sneeze 3 - Individual pigs cough/sneeze 4 - No coughing or sneezing	1	2	3	4	5
4	Conjunctivitis and/or nasal discharge is present.<br>***Underline the appropriate answer***<br><br>0 - More than 30% of pigs have conjunctivitis/nasal discharge 1 - 25% of pigs have conjunctivitis/nasal discharge 2 - 10% of pigs have conjunctivitis/nasal discharge 3 - individual pigs have conjunctivitis/nasal discharge 4 - No pigs no conjunctivitis/nasal discharge	1	2	3	4	5
5	Skin lesions and/or abscesses are present.<br>***Underline the appropriate answer***<br><br>0 - More than 30% of pigs have skin lesions and/or abscesses 1 - 25% of pigs have skin lesions and/or abscesses 2 - 10% of pigs have skin lesions and/or abscesses 3 - individual pigs have skin lesions and/or abscesses 4 - No visible injuries or abscesses	1	2	3	4	5
6	Diarrhea is present.<br>***Underline the appropriate answer***<br><br>0 - More than 30% of animals have diarrhea 1 - 25% of animals have diarrhea 2 - 10% of animals have diarrhea 3 - individual pigs have diarrhea 4 - No visible problems with diarrhea	1	2	3	4	5
7	Umbilical and inguinal hernias are present.<br>***Underline the appropriate answer***<br><br>0 - More than 30% of pigs have umbilical/inguinal hernias 1 - 25% of pigs have umbilical/inguinal hernias 2 - 10% of pigs have umbilical/inguinal hernias 3 - individual pigs have umbilical/inguinal hernias 4 - No pigs have umbilical/inguinal hernias	1	2	3	4	5

8	As you walk through the farm, you notice individual dead pigs.	1	2	3	4	5
	**LIVING CONDITIONS**					
1	The stocking density in the pens is too high (just an observation).<br><br>0 – Stocking density is too high in 30% of pens 1 – Stocking density is too high in 25 % of pens 2 – Stocking density is too high in 10% of pens 3 - Stocking density is too high in individual pens 4 - Pigs have 10% additional area than the minimum necessary	1	2	3	4	5
2	There is enough feeding space and feeders available for the animals.<br><br>0 – Feeders and feeders are not accessible to most animals / there are fewer of them than legally allowed – 1 Water is poured into troughs 2 – In restrictive feeding, there are too few feeding places / Due to the excessive number of animals, there are not enough feeders and feeders / in some pens there are not enough feeders and feeders 3 - According to standards 4 - In each pen there are more than the minimum prescribed controllers and feeders, all animals have access to them	1	2	3	4	5
3	Pigs have enrichment material.<br>***Describe the type of material you see and whether the animals use it.***<br><br>0 – There is no enrichment material, 1 - The material is in individual pens, but the animals do not use it 2 - The material is present, but not in all pens 3 - At least 1 form of enrichment material in each pen (according to standards) is used 4 - Straw in all pens and accessible to all animals	1	2	3	4	5
4	The lighting is visibly inappropriate (too bright, too dark).<br>***Underline the appropriate answer***<br><br>0 - lighting is inadequate in the entire building (too bright/too dark) 1 - more than half of the building is inadequately lit 2 - individual rooms are too bright/too dark 3 - according to standards (lighting is arranged in all rooms), 4 - lighting is measured with meters	1	2	3	4	5
5	Other categories of pigs, even other types of animals, are present in the department.<br><br>0 - All categories of animals are in the same room/ other types of animals are present, 1 - Only one category is separated 2 – The breeding herd is separated from weaners and finishing pigs 3 - Each category is in a separate room 4 - All categories are separated by individual departments, »all in all out« can be performed	1	2	3	4	5
	**ENVIRONMENTAL PARAMETERS**					
1	There are no thermometers and hygrometers or there are too few of them.<br>***Underline the appropriate answer***<br><br>1 - None, 2 - only thermometer/hygrometer 3 - both, but not in every room 4 - every room has a thermometer and hygrometer	1	2	3	4	5
2	There is a lot of dust in the farm.<br><br>0 - The farm is very dusty, the dust can be felt in the air 1 - There is a lot of dust in the farm 2 - There is a moderate amount of dust in the farm 3 - There is little dust in the farm 4 - The farm is completely clean, without dust	1	2	3	4	5
3	There is a lot of humidity in the farm.<br><br>0 - Humidity strongly deviates from the norms in all categories 1 - Humidity deviates from the norms in most categories 2 - Humidity deviates slightly from the norms in certain categories 3 - The humidity in the farm is optimal according to the category 4 - Humidity is in all rooms optimal according to the category and constantly regulated	1	2	3	4	5
4	The smell of ammonia is strongly perceptible in the farm.<br><br>0 - Exceeds the maximum permissible limit (25 ppm) 1 - In more than 50% of the rooms, there is a strongly perceptible smell of ammonia (Above 10 ppm) 2 - In a few categories a stronger smell of ammonia is detected (between 5 - 10 ppm) 3 - A mild smell of ammonia (up to 5 ppm) in the farm 4 - The smell of ammonia is not detectable in any of the rooms	1	2	3	4	5
5	The ventilation is not properly arranged.<br>***Write the method of ventilation.***<br><br>0 - There is no regulated ventilation 1 - Ventilation is regulated, but insufficient 2 - Ventilation is regulated, but in certain categories / rooms insufficient 3 - Ventilation is regulated	1	2	3	4	5

	according to standards (at least natural ventilation present), 4 - Ventilation is regulated and suitable in all rooms					
6	The heating is not properly arranged.<br>***Write the heating method***.<br><br>0 - Heating is not arranged for any category of animals 1 - Heating is arranged, but insufficient in all rooms 2 - Heating is arranged, but insufficient for piglets 3 - Heating is adequately arranged according to standards (at least IR lamps for piglets and floor heating in breeding) 4 - Heating is arranged and regulated by thermometers in all rooms/categories of animals	1	2	3	4	5

PIGS – SPECIAL PART related to the attitude towards their animals (the breeder answers the questionnaire after viewing the farm)

How important do you think it is ... ?

1 not important at all, 2 not important, 3 undecided 4 it is important, 5 it is very important

27		POINTS				
	**GENERAL STATUS**					
1	.. to address the animals when you enter the farm?	1	2	3	4	5
2	... that you have no uninvited guests in the bar nor to record each visit in the entrance/guest book ?	1	2	3	4	5
3	... that the water-trough is not leaking, that the floor of pens is not dirty, that the bedding is fresh?	1	2	3	4	5
4	... that the feed is not contaminated / moldy, that the feed is not scattered on the ground, that the<br>water is clean?	1	2	3	4	5
5	.. that pigs are not dirty, hurt or have bitten tails and/or ears?	1	2	3	4	5
	**ANIMAL BEHAVIOR**					
1	... if the animals are less curious, more aggressive than usual?	1	2	3	4	5
2	... if the animals are scared in your presence, in the presence of a stranger?	1	2	3	4	5
3	... if the animals are huddled at one end of the pen?	1	2	3	4	5
4	... if individual animals have difficulty walking, limp?	1	2	3	4	5
5	... to get to the individual animal as easily as possible and with as little stress as possible for the animal?	1	2	3	4	5
6	Do you observe the behavior of the animals on the farm?	1	2	3	4	5
	**HEALTH STATUS**					
1	... that the animals are equal in size, that there are no cachectic pigs present?	1	2	3	4	5
2	... that the animals do not have damaged trotters and that they do not limp?	1	2	3	4	5
3	... that animals don't cough or sneeze?	1	2	3	4	5
4	... that the animals do not have inflamed conjunctivae or discharge from the nostrils?	1	2	3	4	5
5	... that the animals do not have skin lesions and/or abscesses?	1	2	3	4	5

6	… that pigs do not have diarrhea?	1	2	3	4	5
7	… that pigs do not have umbilical and inguinal hernias?	1	2	3	4	5
8	… that you remove dead animals regularly (once or several times a day)?<br>***Circle how many times***	1	2	3	4	5
	**LIVING CONDITIONS**					
1	... the number of animals per unit area?	1	2	3	4	5
2	... that the animals have enough feeding space and water-throughs?	1	2	3	4	5
3	… that the animals have enrichment materials?<br>***Circle which seem sufficient to the breeder: chains, ropes, balls, bottles, wood, straw***	1	2	3	4	5
4	... that the lighting is not too strong, too weak or too short, too long?<br>***Circle what the breeder thinks is most important: too strong, too weak or too short, too long.***	1	2	3	4	5
5	... that different categories, other types of animals (cattle, chickens...) are not present in the same section?	1	2	3	4	5
	**ENVIRONMENTAL PARAMETERS**					
1	... to know the temperature and humidity in the farm?<br>***Circle what the breeder thinks is more important: temperature or humidity.***	1	2	3	4	5
2	... that there is not a lot of dust in the farm?	1	2	3	4	5
3	… that there is not much moisture in the farm?	1	2	3	4	5
4	... that there are no unpleasant odors in the farm (presence of ammonia)?	1	2	3	4	5
5	... that the ventilation in the stable is regulated?	1	2	3	4	5
6	... that the heating in the stable is arranged?<br>***Circle what the breeder thinks is more important: ventilation, heating.***	1	2	3	4	5
