# Supplementary material for: Discrepancies between farmers' perceptions and actual animal welfare conditions on commercial pig farms
Source: Front Vet Sci. 2022 Sep 29;9:1010791. doi: 10.3389/fvets.2022.1010791 (PMC9558291; doi:10.3389/fvets.2022.1010791)
Supplement: Supplementary file 2 [file Data_Sheet_2.pdf]

Supplementary material S2: Comparisons between observational assessment and self-assessed importance of animal welfare.

Parameters	Observational assessment			Self-assessed importance of welfare			Wilcoxon		Effect size
	M	*SE*	*SD*	*M*	*SE*	*SD*	*Z*	*p*	*r*
General status (A)	***3.7***	***0.16***	***0.60***	***4.6***	***0.09***	***0.35***	***–3.084***	***0.002***	***–0.82***
A – Greeting the animals	3.7	0.19	0.73	4.1	0.33	1.23	–1.140	0.254	–0.30
A – Biosecurity	3.4	0.27	1.02	4.6	0.20	0.74	–2.722	0.006	–0.73
A – Farm hygiene	3.8	0.15	0.58	4.5	0.17	0.65	–2.486	0.013	–0.66
A – Feed and water quality	3.9	0.21	0.77	4.7	0.13	0.47	–2.972	0.003	–0.79
A – Animal condition	3.5	0.25	0.94	4.9	0.07	0.27	–2.980	0.003	–0.80
Animal behavior (B)	***3.9***	***0.12***	***0.46***	***4.4***	***0.11***	***0.42***	***–2.947***	***0.003***	***–0.79***
B – exploratory behavior	3.7	0.19	0.73	4.4	0.20	0.74	–2.714	0.007	–0.73
B – pigs' fear of humans	3.9	0.18	0.66	3.9	0.21	0.77	0.000	1.000	0.00
B – animals crowded	3.9	0.16	0.62	4.2	0.24	0.89	–1.414	0.157	–0.38
B – limping	4.0	0.15	0.55	4.9	0.10	0.36	–3.207	0.001	–0.86
B – animal handling	3.9	0.21	0.77	4.7	0.13	0.47	–2.807	0.005	–0.75
B – observing the animals	4.2	0.19	0.70	4.6	0.14	0.51	–1.890	0.059	–0.51
Health status (C)	***3.8***	***0.08***	***0.30***	***4.8***	***0.07***	***0.25***	***–3.306***	***0.001***	***–0.88***
C – problems with trotters	3.9	0.10	0.36	4.9	0.07	0.27	–3.638	<0.001	–0.97
C – coughing and sneezing	3.8	0.15	0.58	4.7	0.13	0.47	–3.127	0.002	–0.84
C – conjunctivitis and nasal discharge	3.6	0.20	0.74	4.6	0.13	0.50	–3.071	0.002	–0.82
C – skin lesions, abscesses	3.5	0.14	0.52	4.6	0.13	0.50	–3.017	0.003	–0.81
C – diarrhea	4.0	0.10	0.39	5.0	0.00	0.00	–3.500	<0.001	–0.94
C – umbilical, inguinal hernias	4.1	0.07	0.27	4.6	0.13	0.50	–2.828	0.005	–0.76
C – regularity of carcass removal	4.0	0.00	0.00	5.0	0.00	0.00	–3.742	<0.001	–1.00
Living conditions (D)	***3.6***	***0.12***	***0.43***	***4.5***	***0.11***	***0.40***	***–3.188***	***0.001***	***–0.85***
D – stocking density	3.8	0.19	0.70	4.7	0.13	0.47	–2.754	0.006	–0.74
D – feeding space	3.9	0.20	0.73	4.9	0.10	0.36	–2.919	0.004	–0.78
D – enrichment material	3.2	0.21	0.80	4.4	0.20	0.76	–2.846	0.004	–0.76
D – lighting	3.7	0.13	0.47	4.1	0.16	0.62	–1.890	0.059	–0.51
D – separated categories	3.1	0.29	1.10	4.2	0.33	1.25	–2.949	0.003	–0.79

Environmental conditions (E)	***3.7***	***0.09***	***0.32***	***4.3***	***0.13***	***0.50***	***–2.981***	***0.003***	***–0.80***
E – thermometers, hygrometers	2.8	0.19	0.70	4.1	0.27	1.00	–2.970	0.003	–0.79
E – dust	3.8	0.15	0.58	3.9	0.16	0.62	–1.000	0.317	–0.27
E – humidity	4.0	0.00	0.00	4.3	0.19	0.73	–1.414	0.157	–0.38
E – odors	3.7	0.16	0.61	4.5	0.14	0.52	–3.051	0.002	–0.82
E – ventilation	3.9	0.13	0.47	4.4	0.20	0.74	–1.604	0.109	–0.43
E – heating	3.8	0.11	0.43	4.6	0.17	0.65	–3.051	0.002	–0.82

Notes: M = Mean; SE = Standard error, SD = Standard deviation.
