# Supplementary material for: Discrepancies between farmers' perceptions and actual animal welfare conditions on commercial pig farms
Source: Front Vet Sci. 2022 Sep 29;9:1010791. doi: 10.3389/fvets.2022.1010791 (PMC9558291; doi:10.3389/fvets.2022.1010791)
Supplement: Supplementary file 3 [file Data_Sheet_3.pdf]

Supplementary material S3: Observational assessment and self-assessed importance of animal welfare effect sizes for gender, age, education level and sources of vocational training.

**Statistics**	***M***	***SE***	***SD***	***M***	***SE***	***SD***	**Mann-Whitney**		**Effect size**
**A - Gender**	**Males (N = 7)**			**Females (N = 7)**			***Z***	***p***	***r***
O_general status	3.63	0.27	0.71	3.69	0.20	0.53	0.000	1.000	0.00
O_animal behavior	4.14	0.17	0.44	3.71	0.15	0.38	–1.615	0.106	0.43
O_health status	3.97	0.13	0.34	3.70	0.08	0.20	–1.503	0.133	0.40
O_living conditions	3.57	0.22	0.58	3.54	0.09	0.25	–0.129	0.897	–0.03
O_environmental conditions	3.62	0.16	0.42	3.71	0.08	0.21	–0.789	0.430	–0.21
S_general status	4.46	0.14	0.38	4.71	0.11	0.28	–1.447	0.148	0.39
S_animal behavior	4.52	0.17	0.46	4.33	0.14	0.38	–0.709	0.478	–0.19
S_health status	4.84	0.08	0.21	4.66	0.10	0.27	–1.447	0.148	–0.39
S_living conditions	4.43	0.20	0.52	4.49	0.11	0.28	–0.195	0.846	–0.05
S_environmental conditions	4.38	0.21	0.55	4.19	0.18	0.47	–0.901	0.367	–0.24
**B - Age**	**Age ≤ 40 years (N = 4)**			**Age > 40 years (N = 10)**			***Z***	***p***	***r***
O_general status	3.45	0.36	0.72	3.74	0.18	0.57	–0.926	0.354	–0.25
O_animal behavior	3.87	0.21	0.42	3.95	0.16	0.49	–0.358	0.721	–0.10
O_health status	4.00	0.21	0.42	3.76	0.07	0.23	–0.983	0.326	–0.26
O_living conditions	3.45	0.26	0.53	3.60	0.13	0.41	–0.715	0.475	–0.19
O_environmental conditions	3.58	0.11	0.22	3.70	0.11	0.36	–0.800	0.424	–0.21
S_general status	4.60	0.00	0.00	4.58	0.13	0.42	–0.291	0.771	–0.08
S_animal behavior	4.21	0.16	0.32	4.52	0.14	0.43	–1.498	0.134	–0.40
S_health status	4.78	0.11	0.21	4.74	0.09	0.27	–0.073	0.942	–0.02
S_living conditions	4.00	0.12	0.23	4.64	0.09	0.30	–2.729	0.006	–0.73
S_environmental conditions	4.17	0.29	0.58	4.33	0.16	0.49	0.356	0.722	0.10
**C - Education status**	**High school (N = 9)**			**Higher Vocational College or University level (N = 5)**			***Z***	***p***	***r***
O_general status	3.69	0.24	0.71	3.60	0.17	0.37	–0.873	0.382	–0.23
O_animal behavior	3.98	0.17	0.50	3.83	0.18	0.41	–0.539	0.590	–0.14
O_health status	3.75	0.08	0.23	3.98	0.17	0.39	–0.998	0.318	–0.27
O_living conditions	3.62	0.13	0.39	3.44	0.23	0.52	–0.742	0.458	–0.20
O_environmental conditions	3.74	0.10	0.31	3.53	0.14	0.32	–1.029	0.304	–0.27
S_general status	4.56	0.14	0.41	4.64	0.10	0.22	–0.069	0.945	–0.02
S_animal behavior	4.52	0.14	0.41	4.27	0.19	0.42	–1.211	0.226	–0.32
S_health status	4.65	0.09	0.26	4.93	0.05	0.11	–1.922	0.055	–0.51
S_living conditions	4.49	0.13	0.38	4.40	0.22	0.49	–0.542	0.588	–0.14
S_environmental conditions	4.30	0.16	0.48	4.27	0.26	0.59	0.000	1.000	0.00

Statistics	*M*	*SE*	*SD*	*M*	*SE*	*SD*	Mann-Whitney		Effect size
**D - Sources of vocational training**	**Vocational training – two sources (N = 5)**			**Vocational training – more than two sources (N = 9)**			***Z***	***p***	***r***
O_general status	3.24	0.30	0.67	3.89	0.15	0.44	–1.814	0.070	–0.48
O_animal behavior	3.77	0.16	0.35	4.02	0.17	0.50	–1.011	0.312	–0.27
O_health status	3.70	0.12	0.26	3.90	0.10	0.31	–0.499	0.618	–0.13
O_living conditions	3.24	0.07	0.17	3.73	0.15	0.44	–2.225	0.026	–0.59
O_environmental conditions	3.60	0.09	0.19	3.70	0.13	0.38	–0.823	0.411	–0.22
S_general status	4.64	0.13	0.30	4.56	0.13	0.38	–0.343	0.731	–0.09
S_animal behavior	4.20	0.18	0.40	4.56	0.13	0.39	–1.547	0.122	–0.41
S_health status	4.65	0.13	0.30	4.81	0.07	0.22	–1.030	0.303	–0.28
S_living conditions	4.24	0.18	0.41	4.58	0.12	0.37	–1.151	0.250	–0.31
S_environmental conditions	3.80	0.18	0.40	4.56	0.10	0.31	–2.620	0.009	–0.70

Notes: O = observational assessment; S = self-assessed importance of animal welfare; M = Mean; SE = Standard error, SD = Standard deviation.
